# Microbial modulation of host body composition and plasma metabolic profile

**DOI:** 10.1038/s41598-020-63214-1

**Published:** 2020-04-16

**Authors:** M. Nazmul Huda, Jason H. Winnike, Jocelyn M. Crowell, Annalouise O’Connor, Brian J. Bennett

**Affiliations:** 1 Obesity and Metabolism Research Unit, USDA, ARS, Western Human Nutrition Research Center, Davis, California, USA; 2 Department of Nutrition, University of California Davis, Davis, California, USA; 3 David H. Murdock Research Institute (DHRMI), Kannapolis, NC USA; 4 Nutrition Research Institute, University of North Carolina, Chapel Hill, Kannapolis, NC USA

**Keywords:** Microbiome, Genetics research

## Abstract

The gut microbiota is a critical mediator of nutrition and disease risk. Like most complex traits, the microbiome is under genetic regulation and differs between inbred strains of mice. We tested the effect of fecal microbiota transplantation (FMT) on obesity, and plasma glucose. For this study, we collected microbiota from 2 inbred strains of mice which differ in adiposity and glucose tolerance, C57BL/6J and WSB/EiJ. C57BL/6J female mice (n = 18) were first treated with antibiotics for 4 weeks to ablate the microbiota. Following ablation, the mice were transplanted with microbiota from a C57BL/6J or a WSB/EiJ mouse and clinical traits and plasma metabolomic profiles were interrogated at 2- and 4-weeks post-transplantation. Unexpectedly, the mice receiving WSB/EiJ microbiota increased adiposity but decreased plasma glucose. Metabolomic and 16S microbiota profiling indicated broad metabolic changes occurred during and after FMT. Detailed analysis of these interactions demonstrated specific microbiota-host metabolite interactions which may alter disease susceptibility.

## Introduction

Bacteria is heavily colonized in the gastrointestinal tract, often referred to as the gut microbiota (hereafter referred to as microbiota), which can modulate nutritional status, health, and diseases of the host^1^. The diversity and composition of the gut bacteria have been intensely studied, as well as the microbiota’s impact on the health status of the host^2^. The microbiota has been found to be associated with susceptibility to multiple diseases including: obesity^3^, cardiovascular diseases^4^, renal diseases^5^, and metabolic syndrome (MetSyn)^6^, all of which have impact on major public health. For example, 32% of American adults are obese^7^ and nearly 35% have MetSyn^8^ and the prevalence of these diseases are increasing. However, there are still critical gaps in our knowledge regarding how alterations of the microbiota (dysbiosis) can alter metabolism and thus disease susceptibility. A better understanding of the effect of host genetic-microbiota interactions on the composition of the microbiota and disease susceptibility is needed. A holistic view of the metabolic status of an individual, including both microbiota and host genetics, may provide new insights into the underlying mechanisms of pathobiology which may allow us to modulate disease onset, prognosis, and survival^9,10^.

Metabolomic profiling may provide additional insight into the processes affected by specific bacteria or when the composition of the microbiota is altered^11^. It has been shown that the gut microbiota can have a significant effect on plasma metabolic profile, which can modulate host health^12^. Therefore, pairing metabolomics and microbiota analysis may yield important mechanistic insights. For example, trimethylamine N-oxide (TMAO) was identified as a risk factor for cardiovascular disease through a metaorganismal pathway involving the microbiota and diet^13^.

Laboratory mice are often used to investigate both genetic and microbiota to understand the underlying mechanisms of disease risk. There are hundreds of inbred mouse strains which vary in clinical traits and susceptibility to diseases like obesity and MetSyn. Variation in their microbiota may explain part of the disease variation^14–16^. For example, compared to WSB/EiJ mice, C57BL/6J mice have higher fasting blood glucose levels, lower insulin sensitivity, and higher body fat composition^17^, and are susceptible to obesity, cardiovascular disease, and MetSyn. C57BL/6J and WSB/EiJ mice also have different gut microbiota^18^. Therefore, it can be hypothesized that at least part of the elevated plasma glucose and higher body fat in C57BL/6J mice could be improved by fecal microbiota transplantation (FMT) from WSB/EiJ mice.

Recently, fecal microbiota transplantation (FMT) has been used to treat disorders such as *Clostridium difficile* infection^19^, irritable bowel syndrome (IBS)^20^, ulcerative colitis^21^, obesity^22^, and MetSyn^23^. Animal models such as gnotobiotic mice and antibiotic-treated mice have been used successfully to show that FMT can modulate physiological traits such as obesity^22^ and atherosclerosis^24^. In this study, we used 18 C57BL/6J female mice who were treated with antibiotics for 4 weeks and then received FMT either from a C57BL/6J or a WSB/EiJ mouse to identify the gut bacteria associated with body composition and MetSyn risk factors such as plasma glucose and lipid profile. We assessed gut microbiota at baseline, after antibiotics treatment, and at 2- and 4 –week post FMT by using 16S V4 region next-generation sequencing methodology with QIIME2-DADA2 bioinformatics pipeline^25^. For determining the differential microbiota abundance we used ANCOM^26^. We further analyzed plasma metabolic profile at baseline, after antibiotics treatment and 2-week post FMT using two-dimensional gas chromatography time of flight mass spectrometry (GCxGC-TOFMS) and Biocrates AbsoluteIDQ p150 kit to interrogate the effect of gut microbial depletion and recolonization on the plasma metabolomic profile. The association between plasma metabolic profile and phenotypes or gut microbiota were determined by using ANOVA, t-test, principal component (PCA), and Spearman correlation-based statistics.

## Results

### Fecal microbial diversity and composition changes due to microbial depletion and fecal microbiota transplantation (FMT)

**Figure 1:**
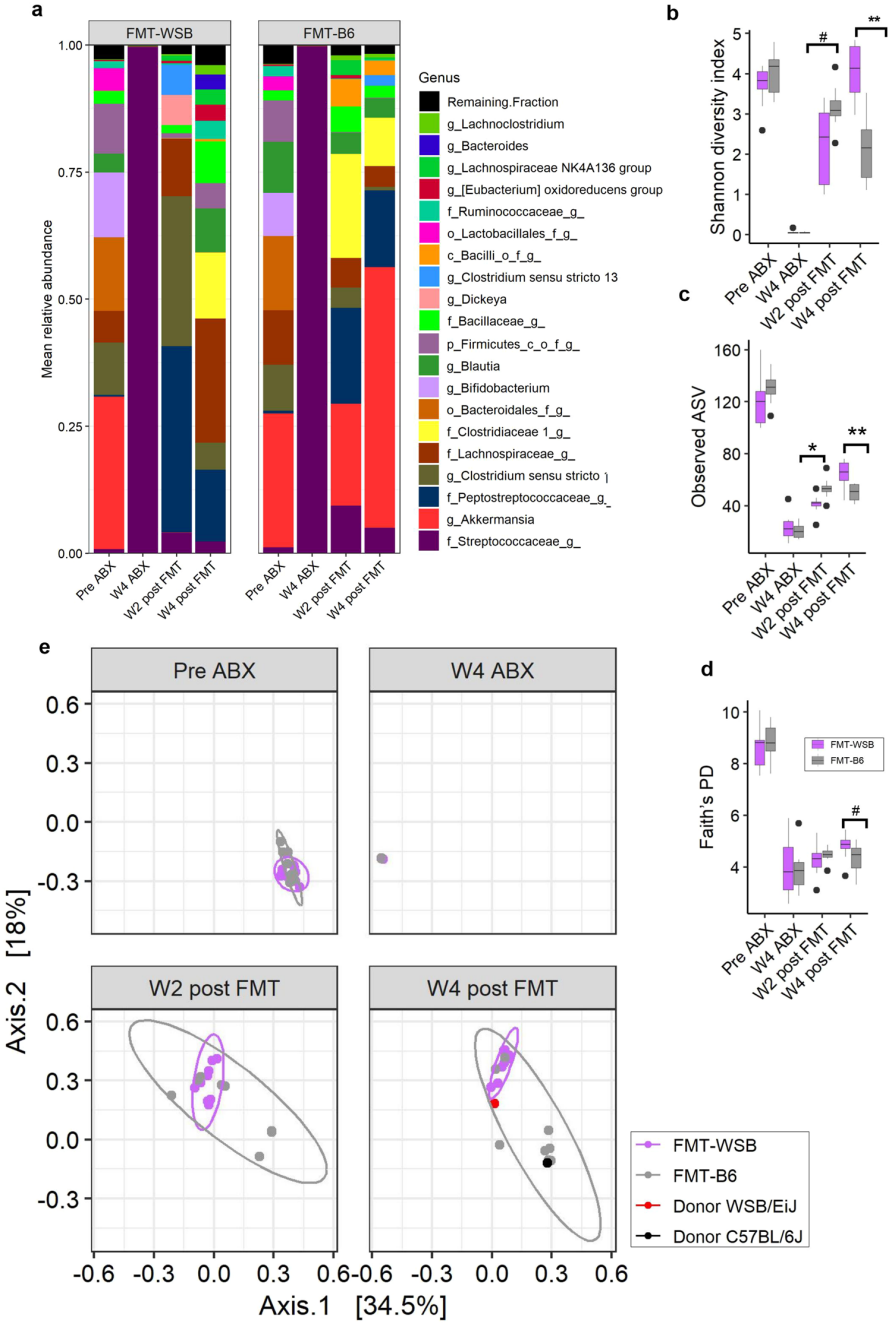
Effect of antibiotics treatment and fecal microbial transplantation (FMT) from a C57BL/6J or a WSB/EiJ mouse on recipient mice’s gut microbiota. (**a**) Mean relative abundance of top 20 genera in mice at baseline, after 4 wk antibiotic treatment, 2 wk, and 4 wk post fecal microbial transplantation by FMT groups. (**b**) Shannon diversity and (**c**) observed ASV, and (**d**) Faith’s Phylogenetic Diversity indices by time points and FMT groups. (**e**) Bray-Curtis beta diversity principal coordinate plot at different time points by the FMT groups. Red and black dot on the 4 wk post fecal transfer plot represents the Bray-Curtis beta diversity measure for the donor WSB/EiJ and C57BL/6J, respectively. The ellipse on the principal coordinate analysis plot indicates 95% CI of the clusters by FMT groups. ** = P < 0.01, # = P < 0.10.

Gut microbiota of eighteen (n = 18) female mice were depleted using an antibiotic cocktail^27^ for 4 wk and then fecal microbiota from a WSB/EiJ or a C57BL/6J mouse was transplanted (Supplemental Fig. 1). Treatment of C57BL/6J mice with antibiotics for 4 weeks caused ablation of almost all the gut bacteria except some bacteria in the family Streptococcacea lineage (Fig. 1a). Fecal microbial transplantation from a C57BL/6J or a WSB/EiJ mice caused a divergence of the microbial composition after transplantation. Bacterial genera related to *Tyzzerella, ASF356, Acetatifactor, Lachnospiraceae UCG-001, Anaerotruncus*, and *Marvinbryantia* lineage from the donner WSB/EiJ mouse did not get colonized in any recipient mouse. The microbiota in the FMT recipient groups (FMT-B6 and FMT-WSB) were similar to their donor microbial composition, but engraftment did not completely replicate the microbiota of the donor strains (Supplemental Fig. 2). Several 16S Amplicon Sequence Variant (ASV) mapping to specific bacterial genera, and families were differentially abundant between FMT-groups as determined by ANCOM at 2 wk (Table 1) and 4 wk post-FMT (Supplemental Table 1). Microbial α-diversity (Shannon diversity index, observed ASV, and Faith’s PD) segregated between FMT groups and observed Amplicon Sequence Variant (ASV) reached statistically significant at 2 wk post-FMT while Shannon diversity index at 4 wk post-FMT (Fig. 1b–d). Both the phylogeny-based (weighted and unweighted UniFrac) and abundance-based (Bray-Curtis) β-diversity were affected by antibiotics treatment and FMT (Fig. 1e and Supplemental Fig. 3). Permutational Multivariate Analysis of Variance (ADONIS) showed that there was a significantly different β-diversity between FMT groups both at 2 wk and 4 wk post-FMT (Table 2).

**Table 1:** Differentially taxa abundance at 2 wk post fecal micrbioal transplantation between mice received C57BL/6J (FMT-B6) or WSB/EiJ (FMT-WSB) fecal microbiota determined at family, genera, and amplicon sequence variance (ASV) levels.

Feature identifier^a^	Family^b^	Genus^b^	W^c^	FMT-B6		FMT-WSB	
				Median (25th, 75th)	Range	Median (25th, 75th)	Range
**Family**							
274a9dbce84cab67bbf98bbd996ae61a	c_Bacilli_o_f_g_		23	459 (295, 882)	147–5327	68 (35.8, 107)	22–976
b7e39d297eeb509ab88cce440a36a83b	f_Akkermansiaceae		25	25200 (9170, 30600)	3–31564	5.5 (3.75, 7.25)	0–27
c5d7b8624f86f1e6c7c74d62753a70c3	f_Lachnospiraceae		20	1940 (1390, 3420)	592–14289	14900 (13000, 18700)	11609–20877
efd8c0105efddba1192866904a26e07b	f_Ruminococcaceae		21	126 (78.3, 221)	39–632	1850(1700, 2090)	1420–2913
**Genus**							
274a9dbce84cab67bbf98bbd996ae61a	c_Bacilli_o_f_g_	c_Bacilli_o_f_g_	43	459 (295, 882)	147–5327	68 (35.8, 107)	22–976
30e43c08a14e7ad72858b1a7157fd01d	f_Ruminococcaceae	g_*Ruminiclostridium 9*	50	0 (0, 6.25)	0–87	201 (104, 295)	43–420
7e22b108e05287de71c88fb1d51ba45b	f_Lachnospiraceae	g_*[Eubacterium] oxidoreducens group*	43	0 (0, 0)	0–21	967 (176, 2020)	0–2993
b7e39d297eeb509ab88cce440a36a83b	f_Akkermansiaceae	g_*Akkermansia*	54	25200 (9170, 30600)	3–31564	5.5 (3.75, 7.25)	0–27
efd8c0105efddba1192866904a26e07b	f_Ruminococcaceae	f_Ruminococcaceae_g_	50	10 (0, 74.25)	0–264	1320 (1260, 1430)	749–1992
**ASV**							
0ac80c6966a22aa4ff1ebc30b86a88ae	f_Clostridiaceae 1	f_Clostridiaceae 1_g_ASV1	236	11.5 (1.5, 16.5)	0–32	0 (0, 0)	0–0
2be344ca421a0e752ea4dc95092a1c47	f_Clostridiaceae 1	f_Clostridiaceae 1_g_ASV2	286	1620 (791, 2400)	28–3108	0 (0, 0)	0–8
2eee016c4e8f0658382ca9a5669d1c62	c_Bacilli_o_f_g_	c_Bacilli_o_f_g_ASV1	287	1890 (976, 2140)	613–3985	0 (0, 2)	0–4
3e9a14f1875c77ada0f2e6e7f8d24d8c	f_Ruminococcaceae	g_*Oscillibacter*	258	114 (32.3, 261)	0–331	0 (0, 0)	0–0
4311be6681becaa0f785e2979b29cee3	f_Clostridiaceae 1	g_*Clostridium sensu stricto 1*_ASV1	267	0 (0, 0)	0–0	1303 (0, 18382)	0–32283
4859baec2549b5610973887db0d9ecf6	c_Bacilli_o_f_g_	c_Bacilli_o_f_g_ASV2	252	25.5 (14.3, 46.5)	0–110	0 (0, 0)	0–0
5475888a6effb097d9cceb292103c327	f_Peptostreptococcaceae	f_Peptostreptococcaceae_g_ASV1	286	0 (0, 0)	0–29	5770 (11, 29300)	0–32298
603e2fef21704715da814484e80a211e	f_Clostridiaceae 1	g_*Clostridium sensu stricto 1*_ASV2	274	561 (153, 1150)	43–1496	0 (0, 17)	0–197
6ae4a0fc0e687ea9f38148b2ac187bbb	f_Streptococcaceae	g_*Lactococcus*_ASV1	244	12 (10, 19)	0–20	0 (0, 0)	0–0
a3ce03f3225ec3cfebf2e3da095170d3	f_Lachnospiraceae	f_Lachnospiraceae_g_ASV1	243	71 (0, 275.25)	0–1389	0 (0, 0)	0–0
b7e39d297eeb509ab88cce440a36a83b	f_Akkermansiaceae	g_*Akkermansia*	238	45.5 (18.3, 19200)	6–21613	1 (1, 6)	0–34
bbaa57cbcd00b123b9132079d19423fe	f_Clostridiaceae 1	f_Clostridiaceae 1_g_	291	4200 (2550, 6600)	131–14144	0 (0, 0)	0–5
c1fd9146929c7f2be99ce0aba2c10ffe	f_Peptostreptococcaceae	f_Peptostreptococcaceae_g_ASV2	289	2190 (1430, 3250)	756–5172	0 (0, 0)	0–2
c5d7b8624f86f1e6c7c74d62753a70c3	f_Lachnospiraceae	g_*Blautia*	285	1090 (401, 2030)	137–4228	2 (0, 5)	0–15
cdef72d7f76f333e18e68e622eff148a	f_Enterobacteriaceae	g_*Dickeya*	240	0 (0, 0)	0–0	66 (0, 1326)	0–2719
d1492ca972b61625ac76fd04962a9b5b	f_Streptococcaceae	g_*Lactococcus*_ASV2	236	0 (0, 0)	0–0	11 (0, 25)	0–36
eb9b3795f8d285b97cec1a0812457398	f_Peptostreptococcaceae	f_Peptostreptococcaceae_g_	254	1330 (464, 3380)	0–7821	0 (0, 0)	0–20
f2d93e5e4d90b71a07acac497854b6ef	f_Lachnospiraceae	f_Lachnospiraceae_g_ASV2	235	58 (0, 155.75)	0–299	0 (0, 0)	0–0

^a^Feature identities equal the MD5 hashes of the 16S rRNA gene sequences. ^b^Maximum available taxonomic information till family or genus level. ^c^W equals the number of ANCOM subhypotheses that have passed for each individual taxon, indicating that the ratios of that taxon’s relative abundance to the relative abundances of W other taxa were detected to be significantly different between two groups. ^d^Differentially abundant taxa was determined by ANCOM at a adj.P value of <0.05.

**Table 2:** Multivariate homogeneity of groups dispersions (betadisper) and Permutational Multivariate Analysis of Variance (ADONIS) analyses of the microbial β-diversity between FMT-B6 and FMT-WSB groups.

	β-dispersion		ADONIS		
	F	P	F.Model	R^2^	P
**Pre-ABX**					
Weighted UniFrac	0.196	0.69	0.242	0.0149	0.86
Unweighted UniFrac	0.512	0.47	1.14	0.0665	0.27
Bray-Curis	0.167	0.66	0.911	0.0539	0.44
**After 4wk-ABX**					
Weighted UniFrac	0.0291	0.84	1.60	0.0965	0.14
Unweighted UniFrac	0.0468	0.82	1.01	0.0633	0.43
Bray-Curis	0.439	0.67	1.51	0.0916	*0.099*
**2wk post FMT**					
Weighted UniFrac	0.0676	0.81	5.20	0.257	<0.001
Unweighted UniFrac	0.649	0.46	3.60	0.194	<0.001
Bray-Curis	2.35	0.13	6.38	0.298	<0.001
**4wk post FMT**					
Weighted UniFrac	0.62	0.48	4.74	0.504	<0.001
Unweighted UniFrac	24.0	<0.001	2.34	0.334	<0.001
Bray-Curtis	2.02	0.16	3.53	0.431	<0.001

### Body composition, plasma glucose and cholesterol are modulated by microbial depletion and recolonization

**Figure 2:**
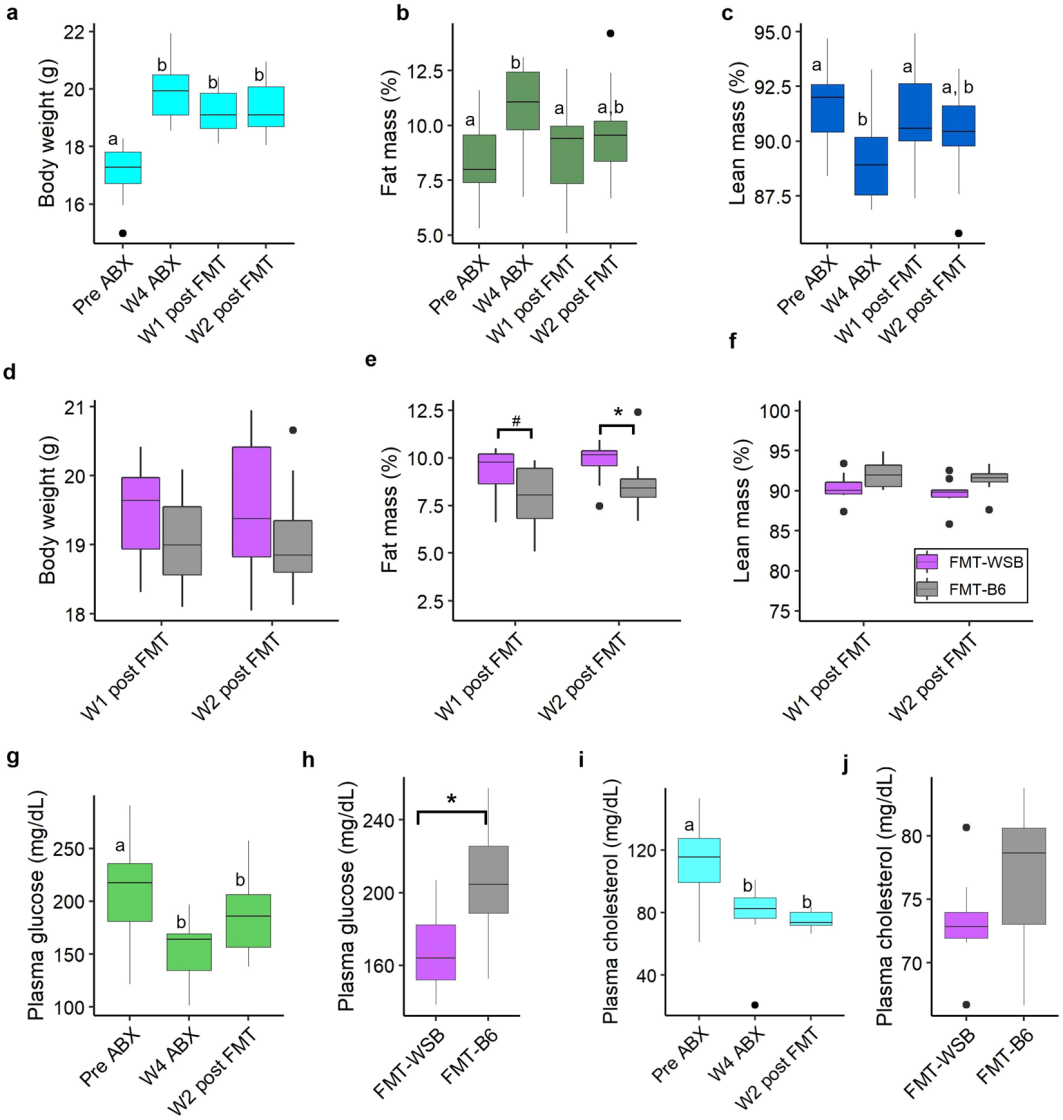
Effect of gut microbial depletion and re-colonization on body composition and plasma clinical biomarkers. (**a**) Body weight (**b**) percent of fat mass, and (**c**) percent of lean mass at baseline, after 4 wk antibiotics treatment, and after 1 and 2 wk post fecal microbiota transplant. (**d**) Body weight, (**e**) percent of fat mass, and (**f**) percent of lean mass at 1 and 2 wk post fecal microbiota transplant by the FMT groups. (**g**) Plasma glucose level at baseline, after 4 wk antibiotics treatment, and after 2 wk post fecal microbiota transplant. (**h**) Comparison of plasma glucose levels at 2 wk post-FMT between FMT groups. (**i**) Plasma cholesterol level at baseline, after 4 wk antibiotics treatment, and after 2 wk post-FMT. (**j**) Comparison of plasma cholesterol levels at 2 wk post-FMT between FMT groups. Boxes with no common letter indicate significant differences. *P < 0.05, #P < 0.10.

To evaluate the effect of microbial depletion and recolonization on the body composition we measured body weight, fat and lean mass by using MRI at baseline, after microbiota depletion, 1 wk post-FMT, and 2 wk post-FMT. We observed a 16.1% increase in mouse body weight (from 17.2 ± 0.8 g to 19.9 ± 1.0 g) after gut microbial depletion (Fig. 2a). No significant difference in the body weight was observed between FMT groups (Fig. 2d). However, there were significant differences in body composition, body fat percentage and lean mass percentage between FMT groups, starting at 2 wk post-FMT (Fig. 2b,c,e,f). FMT-WSB group had an 18.5% higher percent body fat than the FMT-B6 group at 2 wk after fecal transplant. The increased percent body fat was not due to reduced lean mass as we found the lean mass unchanged by FMT, while the fat mass was different between FMT groups (Supplemental Fig. 4).

We then determined if clinical traits related to obesity and MetSyn were influenced by microbial depletion and recolonization with C57BL/6J or WSB/EiJ mouse microbiota. Plasma glucose, cholesterol, and TG were measured at baseline, after microbiota depletion, and 2 wk post-FMT. Plasma glucose and cholesterol levels decreased significantly due to microbial depletion and remained lower throughout the study period (Fig. 2g,i). Mice colonized with WSB/EiJ fecal microbiota were found to have significantly lower plasma glucose levels compared to mice that were recolonized with C57BL/6J fecal microbiota (Fig. 2h). No other plasma clinical chemistry concentrations measured in this study was found responsive to microbial depletion and recolonization by FMT (Supplemental Fig. 5).

### Gut microbiota was associated with body composition and plasma biomarkers

**Figure 3:**
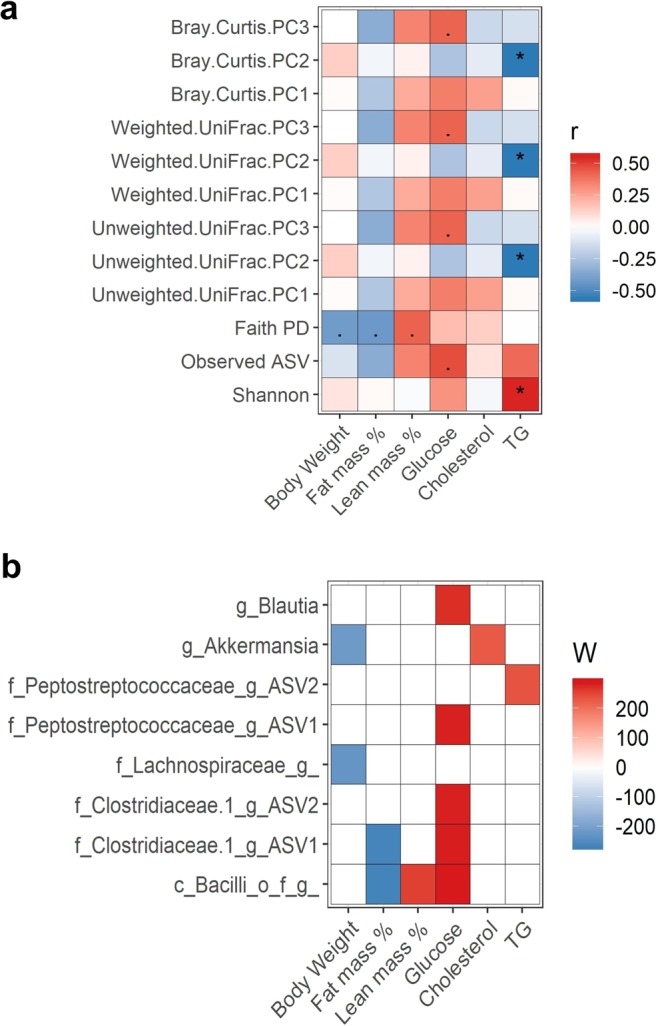
Association between microbiota and phenotypes. (**a**) Spearman correlation between microbial diversity and body composition or plasma clinical parameters at 2 wk post-FMT. “*”P < 0.05, “.”P < 0.10 (**b**) Heatmap showing the ANCOM detected differential bacterial abundance at ASV level between higher (above median) and lower (below median) plasma biochemical parameters at 2 wk FMT. Color key represents ANCOM W value. For easier presentation, ANCOM W values were converted to negative if the mean abundance of the bacteria is lower in the above median group. Red indicates higher ASV abundance in the above median group and blue bacteria represents higher ASV abundance in the below median group. White represents non-significant result obtained  from ANCOM analysis. Red and blue represent significant association determined by ANCOM after FDR correction for multiple comparisons at a significant level adj.P < 0.05.

We performed correlation analysis between gut microbial diversity, body composition and plasma clinical chemistry concentrations. Plasma triglyceride (TG) concentration was positively associated with Shannon diversity index (Fig. 3a). Additionally, several associations between β-diversity indices and plasma TG were observed. To determine which bacterial colonization is associated with body composition and plasma biomarkers at 2 wk post-FMT, we performed ANCOM analysis at ASV level. ANCOM W values were converted to negative values if the mean abundance of the bacteria was lower in the above-median phenotype group. Relative abundances of several bacterial ASV were found to be higher (red) in the higher (above median) phenotype group and several bacterial ASV were found to be lower (blue) in the higher phenotype group (Fig. 3b). For example, *Akkermansia* and Lachnospiraceae abundance at 2 wk post-FMT were found lower in the mice those have higher body weight compared to those have lower body weight. Similarly, two ASVs in the Peptostreptococcaceae family were positively associated with plasma TG and glucose concentrations. Two ASVs in the Clostridiaceae family, one ASV in Bacilli class and *Blautia* were positively associated with plasma glucose.

### Plasma metabolic profile shifted due to microbial depletion and recolonization

**Figure 4:**
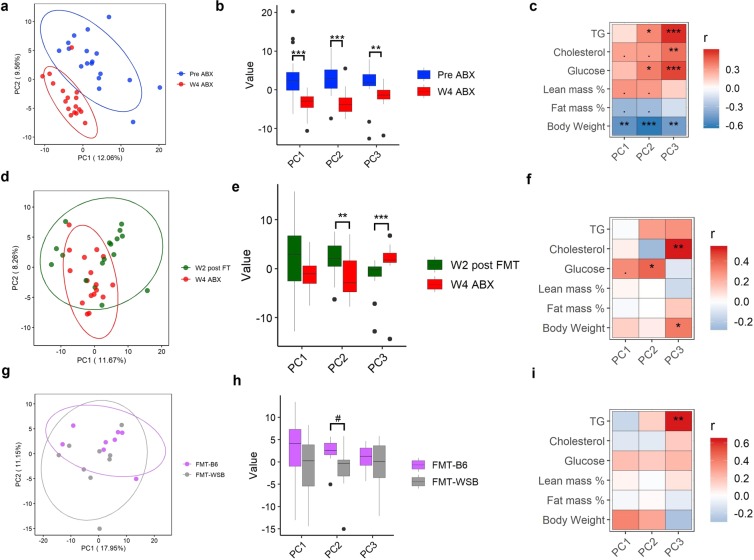
Principal component analysis plot of plasma metabolites. (**a**) Scores are shown for the two first PCs from the PCA of data on plasma metabolites at baseline and after depletion of gut microbiota by 4-wk antibiotics treatment. Each point represents a sample at baseline (blue) or after 4 wk ABX (red). The ellipse on the principal coordinate analysis plot indicates 95% CI of the clusters by study time points. (**b**) Comparison of the contribution of baseline and gut microbiota depleted samples on the PC1, PC2, and PC3. (**c**) Correlation between corresponding PC1, PC2, and PC3 and plasma clinical parameters. (**d**) Scores are shown for the two first PCs from the PCA of data on plasma metabolites after depletion of gut microbiota by 4-wk antibiotics treatment and 2 wk post-FMT. Each point represents a sample after 4 wk ABX (red) or at 2 wk post-FMT (green). The ellipse on the principal coordinate analysis plot indicates 95% CI of the clusters by study time points. (**e**) Comparison of the contribution of baseline and gut microbiota depleted samples on the PC1, PC2, and PC3. (**f**) Correlation between corresponding PC1, PC2, and PC3 and plasma clinical parameters. (**g**) Scores are shown for the two first PCs from the PCA of data on plasma metabolites at 2 wk post fecal microbial transplantation. Each point represents a mouse that received FMT from either a WSB/EiJ (pink) or a C57BL/6J (gray) mouse donor. The ellipse on the principal coordinate analysis plot indicates 95% CI of the clusters by FMT groups. (**h**) Comparison of the corresponding PC1, PC2, and PC3 between samples collected from FMT groups after at 2 wk fecal transplantation. (**i**) Correlation between corresponding PC1, PC2, and PC3 and plasma clinical parameters at 2 wk post-FMT. “***”P < 0.001, “**”P < 0.01, “*”P < 0.05, “.”P < 0.10.

To better understand the underlying metabolic changes in the mice we performed metabolomic analysis at baseline, after microbiota depletion, and 2 wk post-FMT (see methods). The metabolic profile shifted dramatically due to microbial depletion (Fig. 4a,b; ADONIS Bray-Curtis dissimilarity matrix: F.model = 2.80, R^2^ = 0.078, P = 0.049) and recolonization (Fig. 4d,e; ADONIS Bray-Curtis dissimilarity matrix: F.model = 3.22, R^2^ = 0.091, P = 0.044). Principal Components (PCs) of the plasma metabolites showed significant correlation with body composition and plasma biomarkers (Fig. 4c,f). The antibiotic treatment caused 122 metabolites to change significantly from baseline (Supplemental Table 2) and 38 metabolites changed between microbiota depletion and 2 wk post-FMT (Supplemental Table 3). Among the 38 metabolites that changed due to FMT, 18 metabolites (Listed in Supplemental Table 3) decreased significantly after microbial depletion and increased after recolonization by FMT (Supplemental Fig. 6). Additionally, one unknown metabolite (Unknown_RI1082) increased significantly after microbial depletion and decreased significantly after bacterial recolonization (Supplemental Fig. 6 and Supplemental Table 4).

At 2 wk post-FMT, the overall metabolic profile between FMT-B6 and FMT-WSB groups was not significantly different (Fig. 4g,h; ADONIS on Bray-Curtis dissimilarity matrix: F.model = 1.96, R^2^ = 0.115, P = 0.14). We note there was a difference between FMT groups in PC2, (Fig. 4h) and PC3 was correlated to plasma TG level (Fig. 4i). Differential abundance analysis identified 7 plasma metabolites: (3-hydroxyisovaleric acid, methyl-galactoside, ribose, SM C18:1, phenylacetic acid, Lysophosphatidylcholines (LysoPC) a C14:0, and heneicosanoic acid) that were significantly different between FMT-B6 and FMT-WSB groups (Supplemental Table 5). To determine the significance of the metabolites in mice, we then determined the correlation between phenotypes and plasma metabolites. We found 19 metabolites significantly correlated (adj.P < 0.05) with plasma cholesterol, TG and body weight (Supplemental Table 6).

### Specific adiposity related gut microbiota is associated with plasma metabolic profile

**Figure 5:**
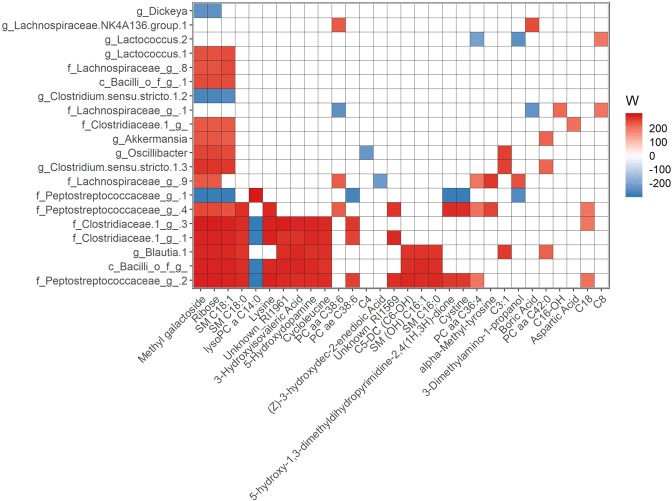
Heatmap showing the ANCOM detected top 20 (based on cumulative ANCOM W value) differential bacterial abundance at ASV level between high (above median) and low (below median) top 30 microbiota associated plasma metabolites at 2 wk FMT. Color key represents ANCOM W value. For easier presentation, ANCOM W values were converted to negative if the mean abundance of the bacteria is lower in the above median group. Red indicates higher ASV abundance in the above median group and blue represents higher ASV abundance in below median group. White color represents non-significant results. Red and blue represent significant association determined by ANCOM after FDR correction for multiple comparisons at a significant level adj.P < 0.05.

To test the differential gut microbiota abundance between mice having high (above median) and low (below median) plasma metabolites concentration we performed ANCOM analysis at baseline and 2 wk post-FMT. At 2wk post-FMT, Clostridiaceae, Peptostreptococcaceae, *Blautia*, and Lachnospiraceae showed significant association with plasma metabolites and were the top 4 influential bacterial taxa. A heatmap of the top 30 plasma metabolites modulated by multiple bacterial taxa and top 20 microbiota modulating multiple metabolites at 2 wk post-FMT is shown in Fig. 5. A complete heatmap with all metabolite - bacteria ASV associations has been depicted in the Supplemental Table 7. At 2 wk post-FMT, a limited number of microbial ASVs were found to be associated with multiple metabolites and similarly a few metabolites were found to be associated with multiple gut bacteria. For example, we identified positive associations between *Akkermansia* and methyl-galactoside, ribose, sphingomyelins (SM) C18:1, phosphatidylcholines (PC) aa C42:0, and linoleic acid. *Akkermansia* abundance was also negatively associated with an unknown metabolite (unknown_RI724) and γ-hydroxybutyric acid (Fig. 5 and Supplemental Table 7). Similarly, methyl-galactoside was found to be positively associated with bacteria related to *Akkermansia, Lactococcus, Oscillibacter, Clostridium sensu stricto 1*, and *Blautia* genus; and Lachnospiraceae, Clostridiaceae, Peptostreptococcaceae, and Clostridiaceae families. Methyl-galactoside was also negatively associated with two ASVs belonging to the genus *Dickeya: Clostridium sensu stricto 1* and *Lactococcus*, and one ASV belonging to the family Peptostreptococcaceae (Fig. 5 and Supplemental Table 7). Among the 19 metabolites significantly associated with plasma TG, cholesterol and body weight (Supplemental Table 6), 13 were found to be associated with differential bacterial ASV abundance (Supplemental Table 8), as determined by ANCOM analysis. There were a limited number of associations between gut bacterial ASV with plasma metabolites at baseline (Supplemental Fig. 7).

## Discussion

The gut microbiota has coevolved with and is now an integral part of mammalian biology^28^. It is well documented that the gut microbiota is associated with obesity^3^, cardiovascular diseases^4^, type-2 diabetes^29^, behavior^30^, immunity^31^, long-lasting vaccine response^32^, and MetSyn^6^. Knowledge about specific mechanistic interactions between gut microbiota, body composition, metabolic health, and metabolic profile is still insufficient. In this study, we have depleted the C57BL/6J mouse gut bacteria using antibiotics and then we recolonized them by FMT from either a C57BL/6J or a WSB/EiJ mouse. These 2 strains are divergent for a number of clinical traits including adiposity and glucose metabolism^17^. We found that gut microbial recolonization with feces from a WSB/EiJ mouse had a profound effect on the body composition, plasma glucose, and cholesterol. We also examined both the plasma metabolome and the gut microbiota to identify interactions mediating these responses.

C57BL/6J mice are more susceptible to obesity, diabetes, and atherosclerosis compared to WSB/EiJ mice^17,18^ and thus often used as models of human diseases. We found that fecal microbial transplantation from a C57BL/6J or WSB/EiJ mouse successfully recolonized in the recipient mice gut and the microbial community started to diverge between FMT recipient groups as evidenced by the α and β -diversity at 2 wk and 4 wk post-FMT. The mice reconstituted with WSB/EiJ microbiota had significantly higher fat mass but lower circulating glucose concentrations compared to the mice reconstituted with C57BL/6J microbiota. Since all the FMT recipient mice (C57BL/6J) in this study were genetically identical, fed the same diet, and similarly housed, the observed higher fat mass in the FMT-WSB group was likely caused by the transplanted gut microbiota. We observed that compared to FMT-WSB group, FMT-B6 group had a significantly higher abundance *of Akkermansia, Blautia*, a bacterium related to class Bacilli lineage, and two bacteria related to the Clostridiaceae family lineage. Our results suggest that there may be a causal inverse relationship between *Akkermansia*, *Blautia*, and adiposity. These bacteria have individually been reported to be associated with changes in adiposity and metabolic parameters. For example, administration of *Akkermansia*, a mucin degrading bacteria, decreased fat mass in diet-induced obese mice^33^. Additionally, *Blautia* decreases with reduced body weight and body fat^34^. How these bacteria interact with each other and other community members remain to be investigated.

In addition to investigating changes in adiposity, we also sought to identify metabolic changes in the FMT mice. Mice reconstituted with WSB/EiJ feces had significantly lower blood glucose levels and a similar trend was observed for plasma cholesterol. Although, the mice reconstituted with WSB/EiJ microbiota are more obese, they are potentially metabolically healthier than mice transplanted with C57BL/6J feces. As noted above several taxa including *Akkermansia*, Peptostreptococcaceae and *Blautia* are differentially abundant between FMT-B6 and FMT-WSB groups. ANCOM analysis identified bacteria in the genus *Blautia*, a bacterium related to class Bacilli lineage, and two bacteria related to the family Clostridiaceae were positively associated with plasma glucose levels in our study. In prediabetes or diabetic humans, *Blautia* abundance was found to be associated with better whole-body insulin sensitivity and lower glucose uptake in the colon^35^. Additionally, we identified *Akkermansia* as higher in mice having above-median plasma cholesterol but was not significantly related to plasma glucose. The data relating *Akkermansia* to plasma glucose and lipids are complex. A recent metagenomic study found *Akkermansia* genes were enriched in type 2 diabetic subjects, however, data from rodents indicate an inverse association between *Akkermansia* abundance and diabetes in NOD mice^36^ and diet-induced obese C57BL/6J mice^33^. Additionally, subjects with MetSyn given supplements of *Akkermansia* have reduced plasma total cholesterol^37^. Overall, our findings, together with previous reports, indicate the complexity of the microbiota in which the relationships between specific bacteria and clinical traits maybe both casual and reactive to disease status.

The complexity of host-microbe interactions is difficult to disentangle. One possible link is the plasma metabolome and thus we focused on metabolic alterations during our FMT protocol. Overall there were dramatic shifts of the plasma metabolites during gut microbial depletion. The microbiota has clear effects on the metabolic profile and germ-free mice have a different plasma metabolic profile compared to conventional mice^12^. Among these microbiota sensitive metabolites, 19 metabolites were significantly modulated by both gut microbiota depletion and recolonization of the microbiota. These metabolites are either synthesized by microbiota or produced by the host in response to the presence of microbiota in the gut. Indeed, among these 19 responsive metabolites, 14 metabolites were found to be associated with specific microbiota at baseline and 7 were associated with specific microbiota at 2 wk post-FMT, indicating potential microbiota-metabolic links. Since our mice were treated with antibiotics, we cannot eliminate the fact that some or all of these altered metabolite levels could be related to direct effects of the antibiotics treatment. In total, we were able to identify a number of metabolites associated with adiposity and metabolic traits.

We next determined the association between gut microbiota and plasma metabolites at 2 wk post-FMT using ANCOM analysis. There were several metabolites associated with specific microbiota. For example, *Akkermansia* was found positively associated with the following metabolites: methyl-galactoside, ribose, SM C18:1, PC aa C42:0, and linoleic acid. Other genus was associated with plasma metabolites such as, *Blautia* whose abundance is associated with methyl-galactoside; ribose; 2-t-butylperoxy-2-ethylbutan-1-ol, propionate ester; 1,5-anhydroglucitol citric acid; docosahexaenoic acid; citrulline; cycloleucine; 5-hydroxydopamine; and 3-hydroxyisovaleric acid. Additionally, *Blautia* abundance was associated with a number of lipid metabolites such as two acylcarnitines C3:1 and C5-DC (C6-OH), 5 sphingolipids (SM (OH) C16:1, SM C16:0, SM C18:0, SM C18:1, and SM C24:1), and 4 phospholipids (PC aa C36:0, PC aa C42:4, PC ae C40:4, PC aa C32:3, PC ae C40:6). *Blautia* is known to expresses enzymes for propionate production^38^ and similar relationships between *Blautia* abundance and propionate has been found in piglets^39^. We also observed that there was a negative associated between *Blautia* abundance and plasma hypotaurine which has been previously reported in Wistar rats^40^. Additionally, we observed that some plasma metabolites were associated with many bacteria at 2wk post-FMT. For examples, we found that methyl-galactoside was positively associated with the following bacteria: *Akkermansia, Blautia, Dickeya*, Bacilli, Peptostreptococcaceae, and Clostridiaceae. Some of these interactions are supported in the literature such as increased methyl-galactoside in patients with increased *Akkermansia* abundance^41^. More importantly, our data suggest that broader changes in community structure may be important to consider when investigating the metabolic consequences of the gut microbiota. Methyl-galactoside itself is an intriguing metabolite as it has anti-fungal activity towards certain fungi^42^. Since gut bacteria and fungi community live together and influence each other^43^, it is highly likely that the alteration of certain fungal abundance can influence a number of gut bacteria and vice versa. Broadening sequenced based approaches to include fungi, viruses, and archaea may aid our understanding of how microbiota influence host phenotype.

We note that in addition to metabolites associated with bacteria, there were several metabolites associated with obesity and dyslipidemia. For example, 2 specific phosphatidylcholines (PC ae C36:4 and PC ae C38:6) were significantly correlated with plasma cholesterol levels which is similar to previous report^44^. Additionally, we found a significant positive correlation between plasma TG levels and a sphingomyelin (SM C24:0) levels which was also supported by a previous study^44^. In our study, we found PC ae C38:6 was positively associated with bacteria related to the Clostridiaceae family and SM C24:0 was positively associated with a bacteria related to the family Lachnospiraceae and Peptostreptococcaceae suggesting a causal relationship between gut microbiota and plasma metabolites which affects clinical traits. However, further mechanistic study is needed to understand the functional consequences of altering these specific phosphatidylcholines and sphingomyelins. We note that our FMT model did not identify all biologically meaningful metabolite:phenotype relationships. For example, numerous studies have found an association between branched-chain amino acids (BCAA) and obesity^45^ but we did not find any such association between BCAA and body fat or body weight in this study. This indicates that the microbiome may not be a significant determinant of this well described pathway and that genetic variants in the host or perhaps disease status itself contributes to this pathway.

Our microbiota depletion protocol included ampicillin, vancomycin, neomycin, and metronidazole^27^ which provides bactericidal activity against both gram-positive and gram-negative bacterial strains. The 4-week antibiotics treatment reduced the bacteria in the feces except few bacteria related to the Streptococcaceae lineage. 16S NGS technique captures DNA from both dead and viable bacteria. Since mouse skin microbiota contains several bacteria^46^, and after oral antibiotics treatment their number decreased but does not abolish completely^47^, it is not surprising that some of these bacterial DNA can be found in the fecal pellets after antibiotic depletion of the gut microbiota. Overall, the decrease in total bacterial DNA and decrease diversity indicate our antibiotic treatment was quite effective.

The growth-promoting effects of sub-therapeutic level low dose antibiotics have been widely used in agriculture^48,49^ to exert selective pressures on gram-positive bacteria to accelerate weight gain by as much as 15%^50^. Although we observed significant changes in body fat percentage during our FMT protocol, the increase in body weight of the mice in the study was similar to large scale phenotyping reported by the Jackson Laboratory^51^. Therefore, the significant increase of body weight during the 4-week antibiotics treatment is likely to be related to normal growth, not a treatment effect. Indeed, previous mouse study also found no changes in body weight due to antibiotic treatment^52^.

In the current study, there was initially a significant decrease in fat mass whereas the lean body mass remained unchanged following FMT. Unlike animals used in agricultural production, laboratory mouse is raised in a pathogen-free environment and thus might have a different microbiota-host relationship which could lead to a difference in the energy requirement for maintaining the already colonized and established gut microbiota^53–55^. The germ-free host gut, after first encountering with gut bacteria, goes through a number of innate and adaptive immune responses which are energy and nutrient expensive^53–55^. Alternatively, the gut of the microbiota depleted mice may have experienced low-grade inflammation during recolonization, which can lower nutrient absorption from food^56^. Once the healthy beneficial bacteria are successfully colonized, they can help harvesting energy from the host diet^57–59^ and thus can contribute a positive energy balance.

Overall, the results of this study highlight the complexity of the metabolic consequences of host-microbe interactions. We observed significant effects of FMT on increases in plasma glucose but unexpected adiposity. Our study utilized samples isolated from mice susceptible and resistant to obesity. As FMT has been proposed to be a potential therapeutic treatment for obesity and Metabolic Syndrome, our results suggest that is complex and thus need more integrative mechanistic research to understand the underlying potential beneficial and harmful health outcomes of FMT.

## Methodology

### Study design

Eighteen (n = 18) four-week-old C57BL/6J female mice were purchased from Jackson Laboratories at (Bar Harbor, ME, USA), and acclimated for 1 week. After the acclimation period, average water consumption was measured for a 7-day period and then mice were subjected to a 4-week gut microbial depletion using antibiotic cocktails, fecal microbiota transplantation (FMT) from either a C57BL/6J or a WSB/EiJ, and 4-week follow-up. For microbiota depletion, mice were given water supplemented with antibiotics for 4 weeks based on consumption^27^. Water flasks were supplemented with 1 g/l ampicillin, 5 mg/ml vancomycin, 10 mg/ml neomycin, and 10 mg/ml metronidazole. The fresh antibiotic cocktail was mixed every day and ampicillin and water was renewed every 7^th^ day. The study procedures are detailed in the Supplemental Fig. 1. Body composition (body weight, fat mass, and lean mass) was measured using an EchoMRITM-100H (Echo MRI LLC, Houston, TX, USA) at 4 time points: baseline (study week 0), after microbiota depletion (study week 4), 1 wk post-FMT (study wk 5), and 2 wk post-FMT (study wk 6) as shown in Supplemental Fig. 1. From each mouse, blood and fresh fecal samples were collected at baseline, after 4 weeks of antibiotics treatment, and 2-week post-FMT. An additional fecal sample was collected at 4-week post-FMT. Fecal samples were stored at −80 °C until further processing. Plasma was separated from blood and stored at −80 °C until further analysis. For the entire study period, mice were group-housed in metabolic cages at 3 mice per cage and standard conditions (12 h light: dark, temperature- and humidity-controlled conditions). Mice were on a nutritionally complete purified synthetic diet containing 9.4% kcal from fat, 75.9% kcal from carbohydrate and 14.7% kcal from protein (AIN93M; #D10012M; Research Diets Inc., New Brunswick, NJ, USA). All experiments were approved by the Institutional Animal Care and Use Committee (IACUC) at the North Carolina Research Campus (NCRC) and the experiment were carried out in accordance with the relevant guidelines and regulations.

### Fecal microbiota transplantation (FMT)

Prior to transplant studies, feces from age and sex-matched donor C57BL/6J and WSB/EiJ mice fed an AIN-93M diet were collected and stored at −80 °C until further use. On the day of inoculation, frozen feces were pulverized with dry ice-cooled mortar and pestle. Fecal powder was suspended in sterile PBS (100 g feces/1000 ml of sterile PBS). The suspended feces were kept on ice and each study mouse was administered 100 μl via oral gavage and transferred to a new clean cage with fresh food and water.

### Plasma clinical biomarkers assay

Mice were fasted for 4 hours before blood draw via retro-orbital bleed. Blood was collected into EDTA-containing tubes and plasma was separated by centrifugation at 10,000 xg for 10 min at 4 °C. Plasma triacylglycerol (TG), total cholesterol, and glucose were measured by Biolis 24i Analyzer (Carolina Liquid Chemistries, Winston-Salem, NC).

### Plasma metabolite assay

Plasma metabolite analysis was performed using two platforms, two-dimensional gas chromatography time of flight mass spectrometry GCxGC-TOFMS (LECO, MI, USA) and Biocrates AbsoluteIDQ p150 kit (Biocrates, Innsbruck, Austria). Data from the two analytical platforms were combined for the downstream analysis. Detailed analysis procedures can be found in the Supplemental Method section.

### Microbiota analysis

Fecal microbiota was analyzed by 16S rRNA V4 sequencing methodology. In brief, total fecal DNA was extracted using ZymoBIOMICS™ 96 MagBead DNA kit (Zymo Research, Irvine, CA) with automated epMotion (Eppendorf, Hamburg, Germany) robotic system. Mixed template amplicon library was prepared according to the protocol from Earth Microbiome Project (http://www.earthmicro biome.org/emp-standard-protocols/) form extracted fecal total DNA using the primer sets (515 F and barcoded 806 R)^60^. The PCR master mix, primer, and samples were plated in triplicate using automated epMotion robotic system (Eppendorf, Hamburg, Germany). The PCR composition and the reaction cycle for the amplicon library preparation has been previously described^31^. Amplicon DNA was multiplexed and sequenced using the Illumina MiSEQ platform with 2 × 250 bp paired-end sequencing. Obtained sequence data were de-multiplexed and analyzed using the open-source software QIIME2-DADA2 pipeline^25^. Taxonomy was assigned using the SILVA 132 reference database^61^ customized for QIIME2 for 16S  V4 (515 F/806 R) region of sequences at the threshold of 99% pairwise identity. A detailed analysis has been described in the supplemental methodology.

### Statistical analysis

Statistical analyses were performed using R version v3.5.1 for Windows^62^. Metadata continuous variables were analyzed for Normality using Shapiro-Wilk normality test and QQ-normal plot. Variables with a Shapiro-Wilk W value ≥0.95 were considered as Normal. Non-Normal metadata variables were transformed by natural log, square root, square, or Box-Cox power transformation. If no appropriate transformation was found, the variables were normalized rank-transformed. Differences of homogeneity of microbial composition dispersions between FMT groups were determined by using PERMDISP2 function of R Package Vegan^63^ with 999 permutations. Differences in microbial community β-diversity were tested by ADONIS (perMANOVA) in the R Package Vegan. Principal coordinate (PCoA) analysis was carried out by PhyloSeq^64^. Differential microbiota abundance was analyzed by ANCOM^26^ using R package ancom.R with default settings and FDR correction.

We measured 354 measured metabolites by GCxGCMS and 163 by Biocrates. Metabolite data were checked for excessive missing values. 56 metabolites measured by GCxGCMS were removed due to a higher number of missing values detected by using R package WGCNA’s^65^ “goodSampleGenes” test. Principal component analysis (PCA) was performed by using R function “prcomp”. The similarities of the metabolites at different study time points or between FMT groups were carried out by PerMANOVA (ADONIS) analysis on Bray–Curtis distance matrix using the vegan package in R^63^. Correlation analysis was carried out by Spearman correlation. Two group comparisons were carried out by two-sample t-tests or Wilcoxon rank-sum tests. Multiple group comparisons were carried out by ANOVA. All P values reported in the study were from two-tailed tests. P values were corrected for multiple comparisons using Benjamini-Hochberg (BH) procedure. P values < 0.05 were accepted as significant for clinical data analysis, and BH adjusted P values (q value) <0.05 was considered as significant for metabolomics and microbiota data. Graphs were prepared by GGplot2^66^ and GraphPad Prism (GraphPad Software, Inc., CA, U.S.A.).

## Supplementary information

Supplemental method and Supplemental Figures.

Supplemental Tables.

## Data Availability

The 16 S sequence data is available at NCBI Sequence Read Archive (SRA) database under the BioProject ID “PRJNA575555”. The phenotype and metabolomics data of this study are available from the corresponding author upon request.
