# Supplementary material for: Microbial modulation of host body composition and plasma metabolic profile
Source: Sci Rep. 2020 Apr 16;10:6545. doi: 10.1038/s41598-020-63214-1 (PMC7162933; doi:10.1038/s41598-020-63214-1)
Supplement: Supplementary file 1 — Supplemental method and Supplemental Figures. [file 41598_2020_63214_MOESM1_ESM.docx]

**Supplemental information – Supplemental Method and Supplemental Figures**

**Manuscript title**: Microbial modulation of host body composition and plasma metabolic profile

**Authors**: M. Nazmul Huda, Jason H. Winnike, Jocelyn M. Crowell, Annalouise O’Connor,

and Brian J. Bennett

**Supplemental Method**

**Plasma metabolite assay**: For plasma metabolite analysis we used two-dimensional gas chromatography time of flight mass spectrometry (GCxGC-TOFMS) and Biocrates AbsoluteIDQ p180 Kit. For GCxGC-MS analysis, metabolites were extracted from 40 µl of plasma using CH3Cl-CH3OH. The resulting supernatant was dried under a gentle flow of N_2_ and derivatized using methoxyamine and N-Trimethylsilyl-N-methyl trifluoroacetamide + chlorotrimethylsilane (MSTFA + TMCS). Processed plasma samples were analyzed on a LECO Pegasus 4D GCxGC-TOFMS (LECO Corporation, MI, USA) with a 60 m DB-5MS-UI (250 µm ID, 0.25 μm film) primary column and a 1 m DB-17MS (250 µm ID, 0.25 μm film) secondary column. Helium (99.9999%) was used as the carrier gas and the flow was constant at 1 mL/min. The inlet temperature was 270°C, the transfer interface temperature was 280°C. The primary oven temperature was programed as follows: oven temperature 70°C for 1 min; increment at +5°C/min up to 280°C and hold for 8.0 minutes. The secondary oven temperature was program as identical to the main oven but with an offset of +5°C above the main oven temperature. The modulator temperature offset above the main oven was +15°C. Mass scans were carried out at a rate of 200 spectra per second. Pooled sample quality control was run every 10^th^ sample throughout the sample sequence. Metabolites (identified or unknown) had to be present in at least half of the QC samples with a relative standard deviation of less than 30% to be included in the final data set. LECO ChromaTOF software was used for spectrum deconvolution and metabolite identification; MetPP ^1^ was used for retention index matching, cross-sample peak list alignment, and metabolite quantification. For Biocrates analysis, we used an AbsoluteIDQ p150 kit. Samples were processed according to manufacturer’s instructions and run on a Sciex 4000 QTRAP with an Agilent 1200 HPLC. Data from the two analytical platforms were combined for the downstream analysis.

**Microbiota analysis**: Total stool DNA was extracted using ZymoBIOMICS™ 96 MagBead DNA kit (Zymo Research, Irvine, CA) with an automated epMotion (Eppendorf, Hamburg, Germany) robotic system. 100 to 150mg of stool samples were placed in the ZR BashingBead™ Lysis Tube and homogenized using FastPerp24 bead beater (Millipore, Hayward, CA) at 6.5 HZ for 2 min. The lysate was centrifuged at ≥10,000xg for 1min and 200μl supernatant from lysis tube were transferred to 96 deep-well plate (Eppendorf, Hamburg, Germany) and loaded in an epMotion 5075t robotic system. Using epMotion, 600μl ZymoBIOMICS™ MagBinding Buffer and 25μl of ZymoBIOMICS™ MagBinding Beads were added to each well, and was mixed well. After mixing the plate was placed on a magnetic stand and the supernatant was discarded. MagBinding Beads were washed with MagWash 1 and MagWash 2 and the DNA was eluted using 50 μl ZymoBIOMICS™ DNase/RNase free water. The DNA concentration was measured using NanoDrop One (Thermo Scientific, Petaluma, CA).

Mixed template amplicon library for the 16S variable region 4 was prepared according to the protocol from Earth Microbiome Project (http://www.earthmicro biome.org/emp-standard-protocols/) using the extracted stool total DNA and the primer sets (515F and barcoded 806R)^2^. The PCR master mix, primer and samples were plated using an automated epMotion robotic system (Eppendorf, Hamburg, Germany). Appropriate NTC, extraction control and pooled fecal sample were added to each plate. The PCR composition and the reaction cycle for the amplicon library preparation has been previously described ^3^. In brief, a master mix was prepared that consisting of 37.5µl of GoTaq Green Master Mix (Promega, Madison, WI), 3µl of 25mM MgCl_2_, 1.5µl of 10µM forward primer 515F, and 25.5 µL of nuclease-free water. Then 1.5µl of 10µM barcode specific reverse primer 806R and 6µl of extracted stool DNA were added. PCR was performed in triplicates of 25µL under the following conditions: denaturation (1 cycle) at 94°C for 3 min; amplification of 25 cycles at 94°C for 45 s, 50°C for 60s, and 72°C for 90s; and a final extension step cycle at 72°C for 10min. Amplicon DNA was multiplexed and purified using Wizard SV Gel and PCR Clean-Up System (Promega, Madison, WI). The amplicon library was sequenced using the Illumina MiSEQ platform with 2x250bp paired-end sequencing. Obtained sequences were de-multiplexed and amplicon sequence variance (ASV) was determined using the open-source software QIIME2-DADA2 pipeline ^4^. A total of 10,133,403 sequences with an average of 116,476 ± 39,525 (mean ± SD) sequences per sample found after demultiplexing were entered into DADA2 analysis. After DADA2 process 85,886 ± 29,458 non-chimeric sequences per sample were survived and constitute the ASV table. Taxonomy was assigned using the SILVA 132 reference database ^5^ customized for QIIME2 for 16s V4 (515F/806R) region of sequences at the threshold of 99% pairwise identity. ASV belonging to mitochondria and chloroplast were filtered out form the initial ASV table. We performed a single rarefaction at a sequence depth of 38,000 sequences per sample. α-diversity (Shannon diversity index, observed species, and Faith’s PD) and β-diversity (unweighted UniFrac, weighted UniFrac, and Bray Curtis) were calculated from the unfiltered OTU table. The OTU table was filtered by removing any OTU present in fewer than 5 samples and with a relative abundance across all samples ≤0.005% to calculate differential bacteria abundance.

**Supplemental Figures**

**
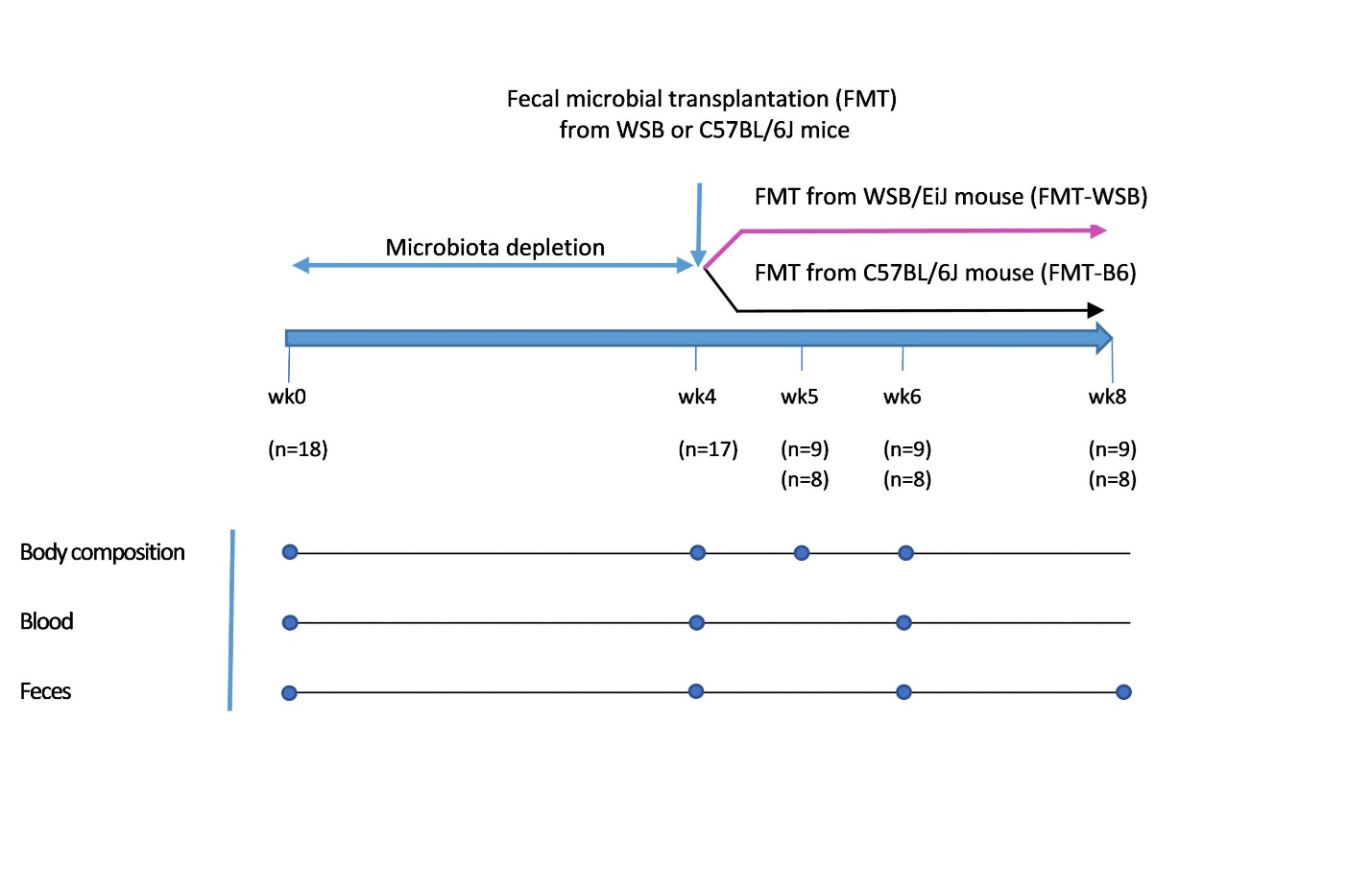
**

**Supplemental Figure 1**: Study design: C57BL/6J female mice (n=18) were treated with antibiotic cocktails for 4 weeks and then received FMT either form a C57BL/6J or WSB/EiJ mice as indicated. Mice were evaluated for 4 weeks after FMT. Each dot represents a corresponding anthropometric data or sample collection event as indicated on the left side of the figure.

**
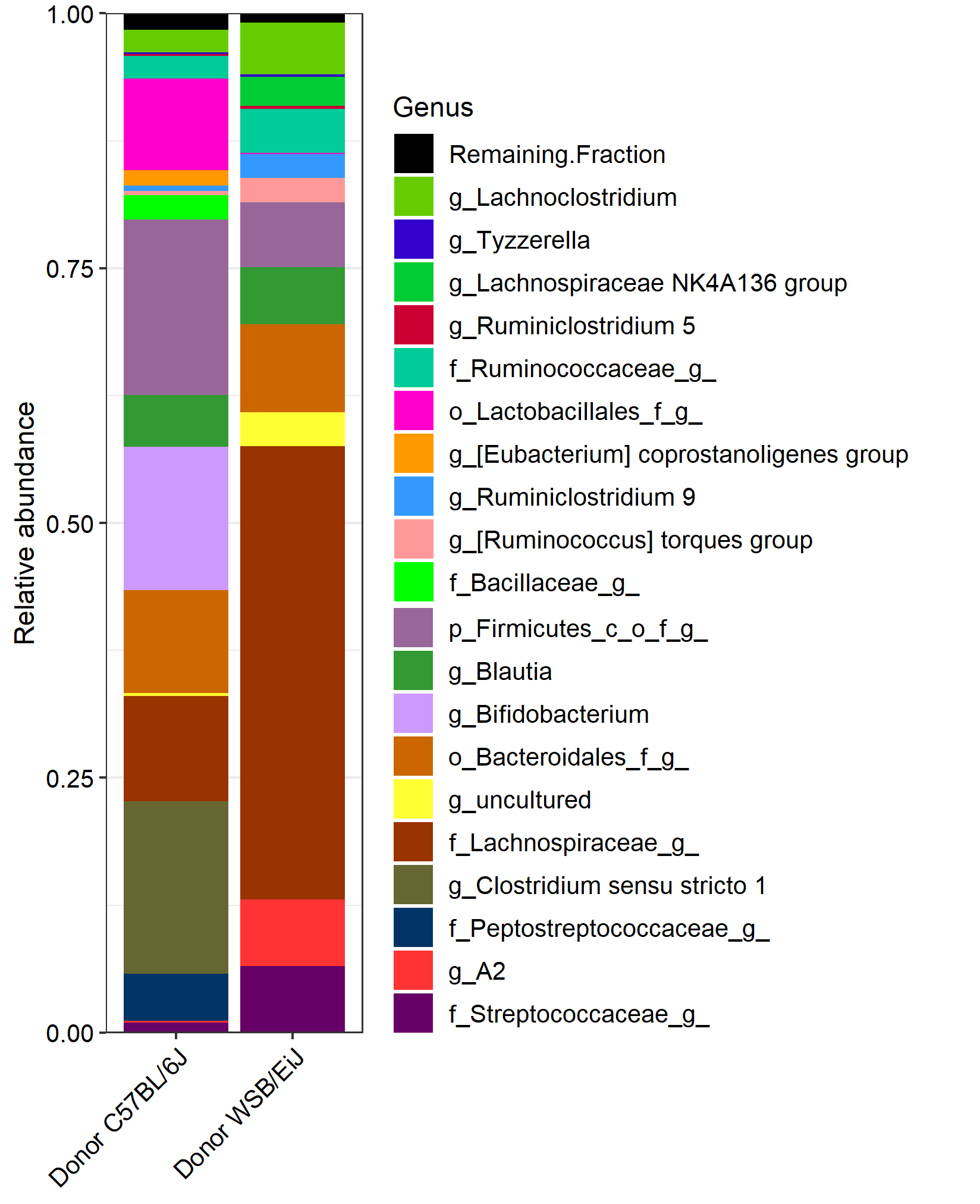
**

**Supplemental Figure 2**: Relative abundance of the top 20 genera in WSB/EiJ and C57BL/6J stool used for fecal microbial transplantation.


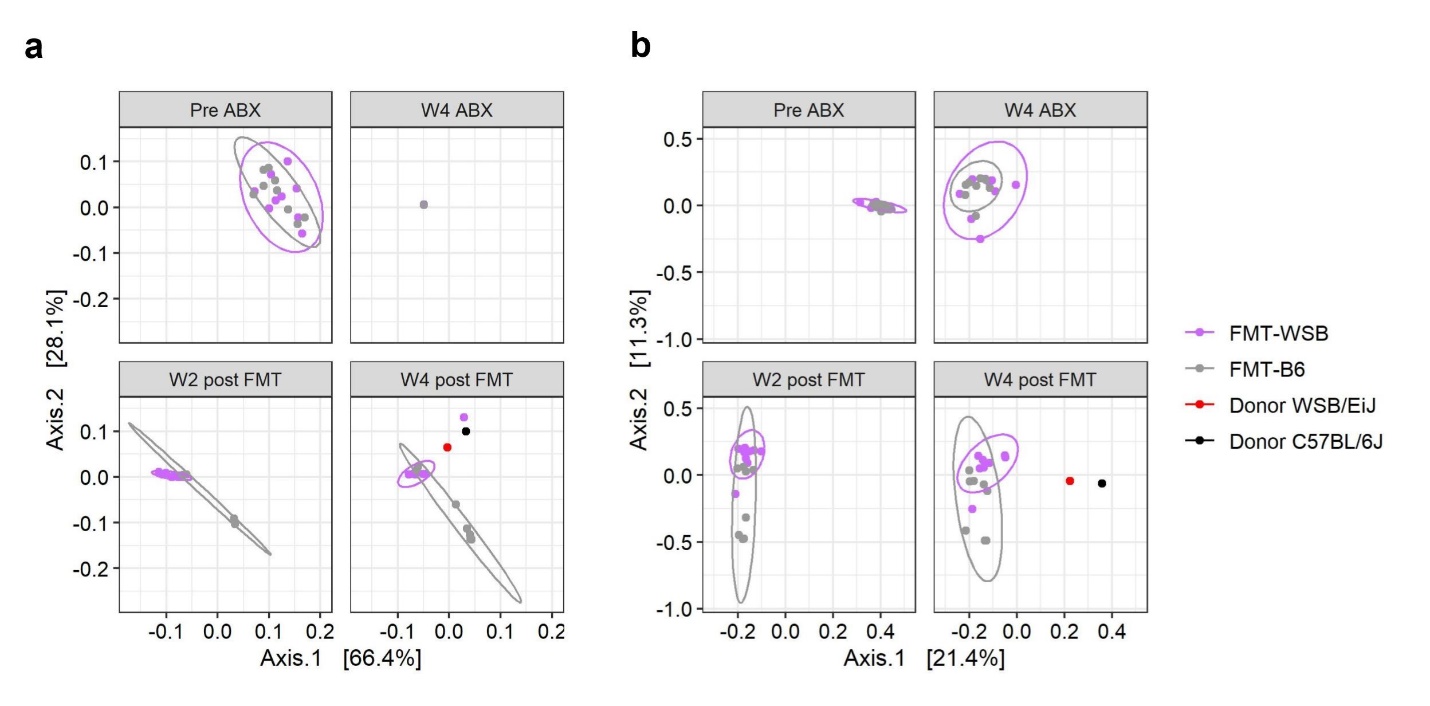


**Supplemental Figure 3**: Effect of antibiotics treatment and FMT on recipient mice’s gut microbial beta diversity. (**a**) Weighted and (**b**) unweighted UniFrac beta diversity principal coordinate plot at different time points by the FMT groups. Red and black dots on the 4 wk post fecal transfer plot represents the corresponding beta diversity measure for the donor WSB/EiJ and C57BL/6J fecal samples, respectively. The ellipse on the principal coordinate analysis plot indicates 95% CI of the clusters by FMT groups.


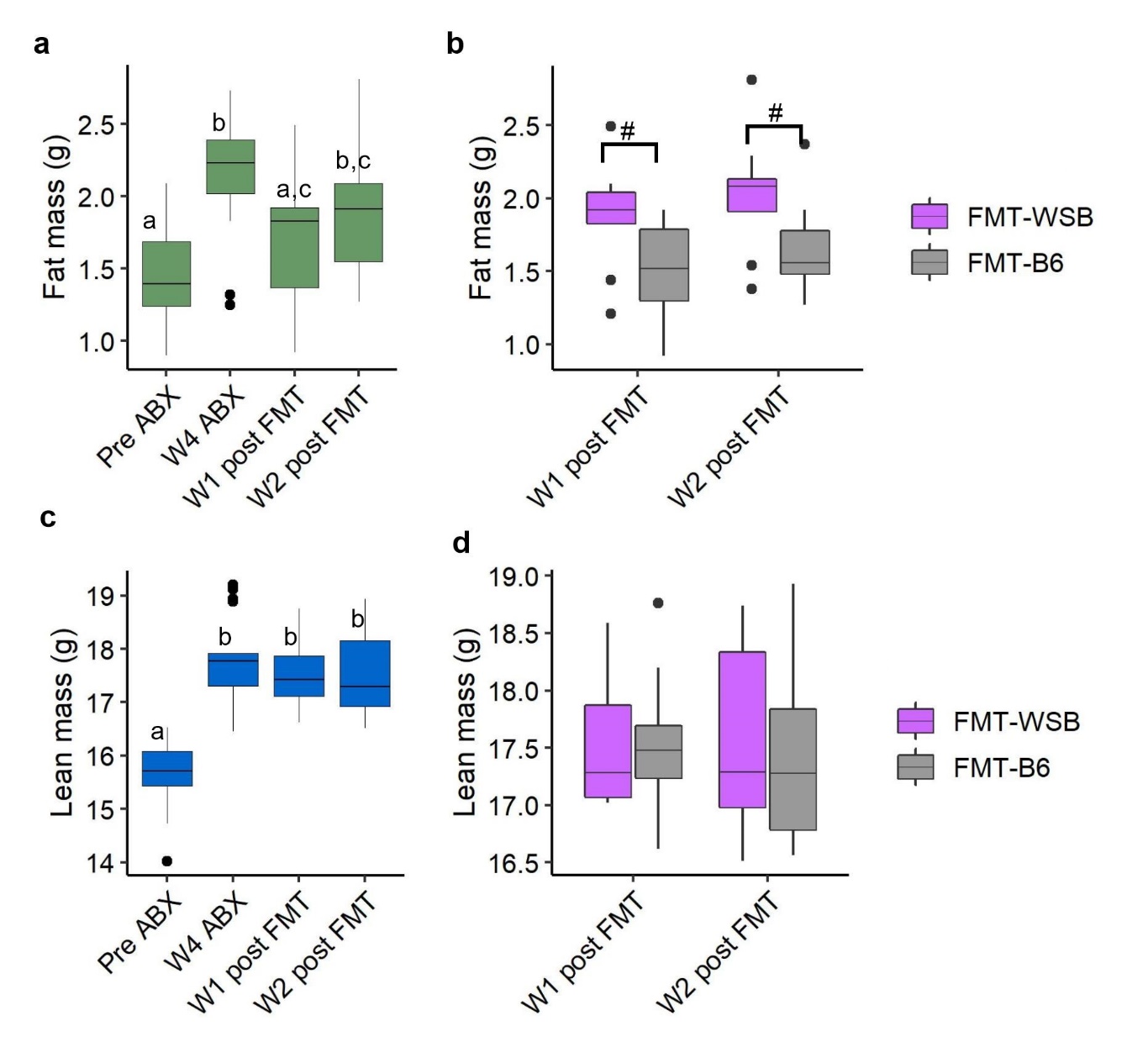


**Supplemental Figure** **4**: Effect of gut microbial depletion and re-colonization on fat and lean mass. (**a**) Fat mass at baseline, after 4 w antibiotics treatment, and after 1 and 2 post fecal microbiota transplant. (**b**) Fat mass at 1 and 2 wk post fecal microbiota transplant by the FMT groups. (**c**) Lean mass at baseline, after 4 wk antibiotics treatment, and after 1 and 2 wk post fecal microbiota transplant. (**d**) Lean mass at 1 and 2 wk post fecal microbiota transplant by the FMT groups. Boxes with no common letter indicate significant differences. # = p<0.10.


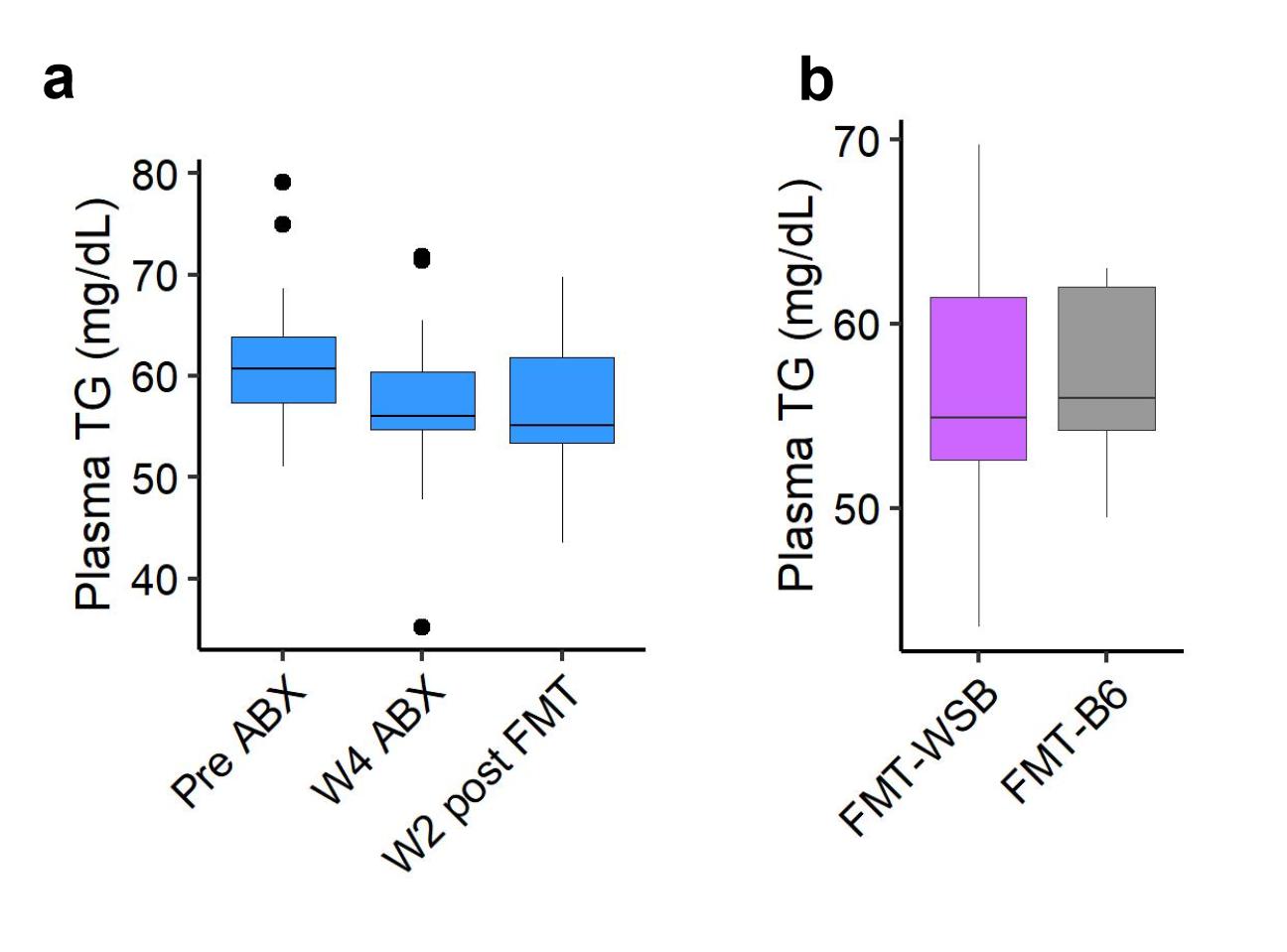


**Supplemental Figure 5**: Effect of gut microbial depletion and re-colonization on plasma TG level. (**a**) Plasma TG level at baseline, after 4 wk antibiotics treatment, and after 2 wk post fecal microbiota transplant. (**b**) Comparison of plasma TG levels at 2 wk post fecal microbiota transplant between FMT groups.

**
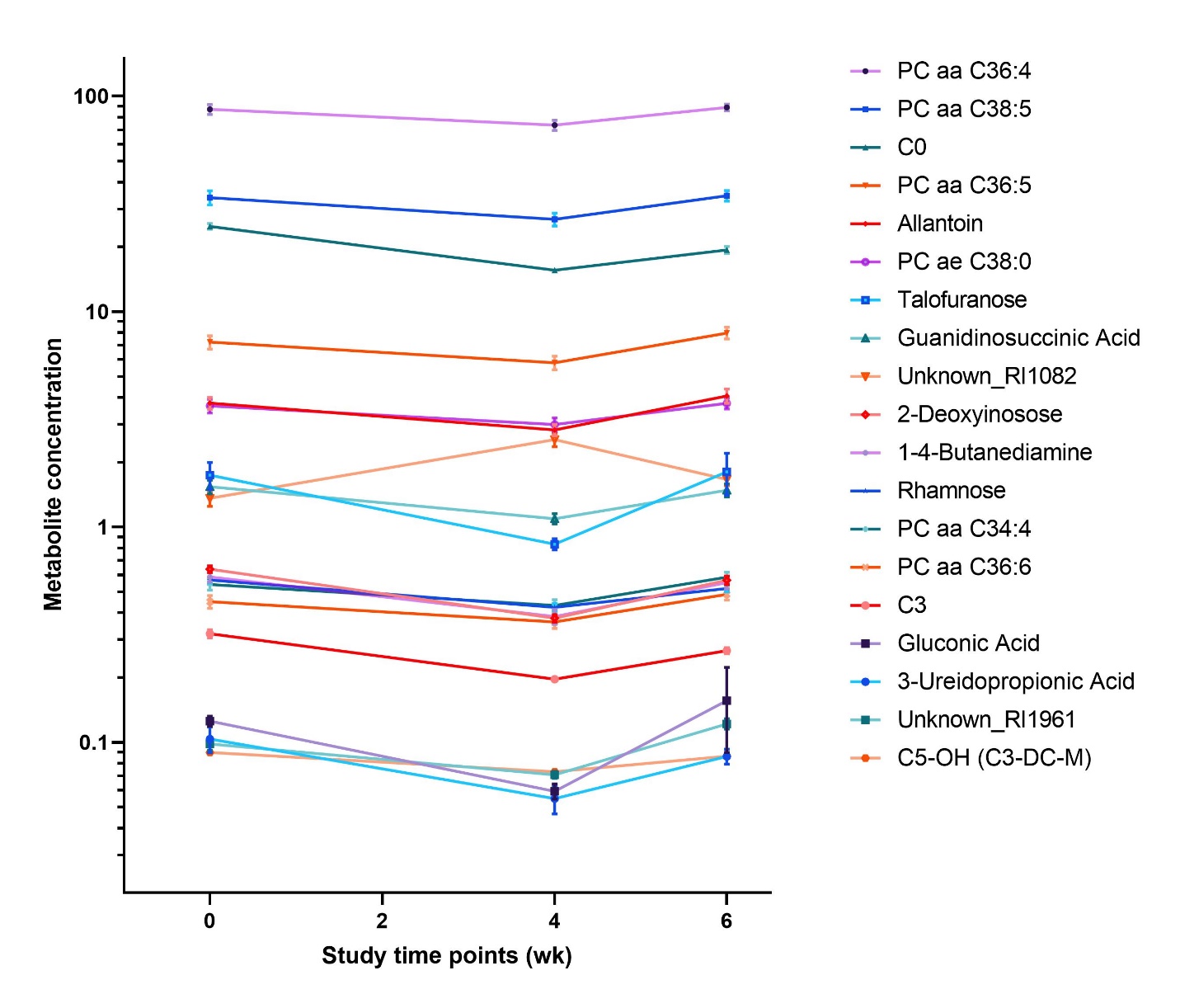
**

**Supplemental Figure 6**: Concentration of 19 microbiota responsive plasma metabolites concentration at baseline, after microbiota depletion, and at 2 wk post-FMT altered significantly by microbiota depletion and recolonization. Values are presented as mean ± SD.


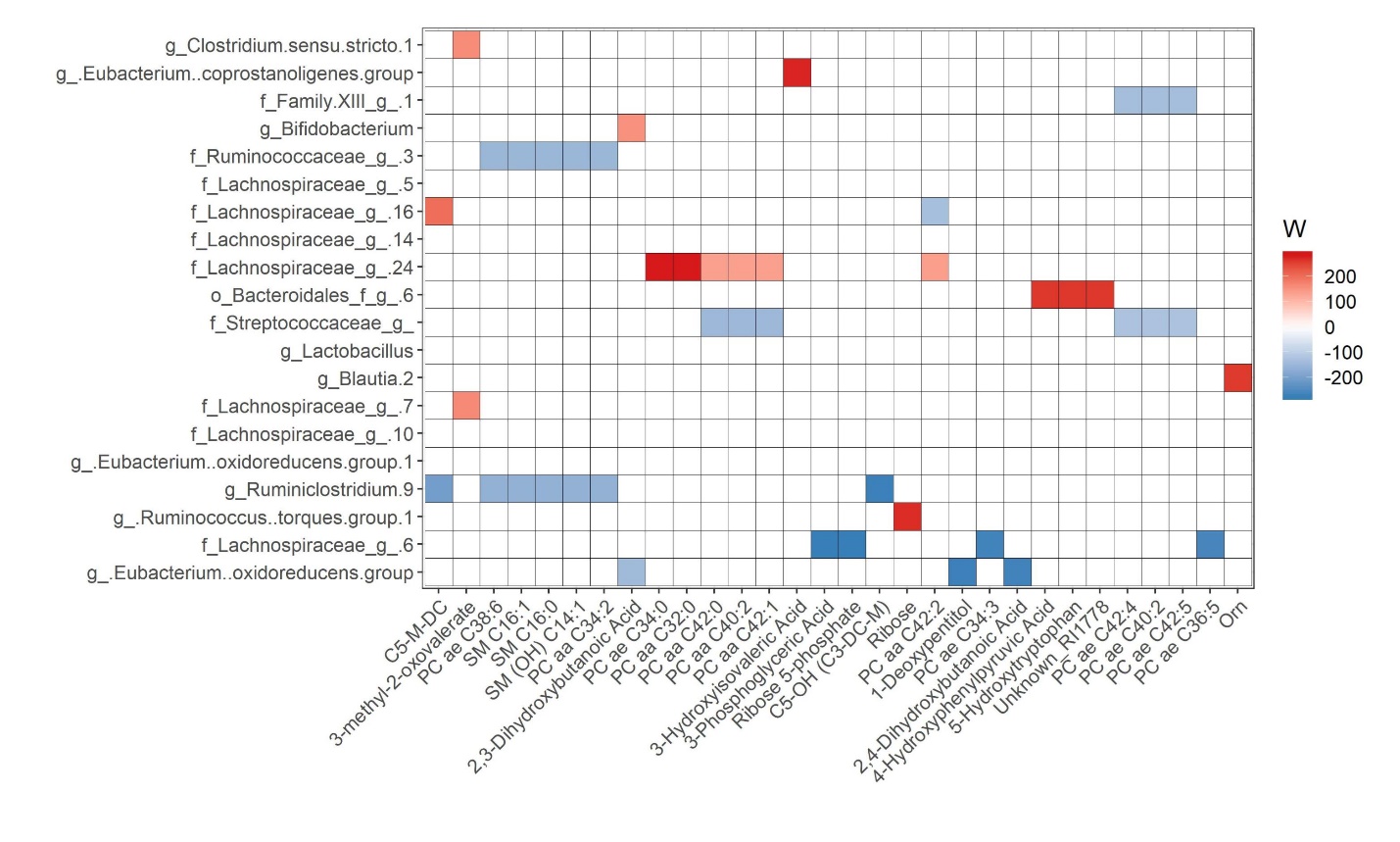


**Supplemental Figure 7**: Heatmap showing the ANCOM detected top 20 (based on cumulative ANCOM W value) differential bacterial abundance at ASV level between high (above median) and low (below median) top 30 microbiota associated plasma metabolites at baseline. Color key represents ANCOM W value. For easier presentation, ANCOM W values were converted to negative if the mean abundance of the bacteria is lower in the above median group. Red indicates higher ASV abundance in the above median group and blue represents higher ASV abundance in below median group. White color represents non-significant results. Red and blue represent significant association determined by ANCOM after FDR correction for multiple comparisons at a significant level adj.P<0.05.

**Supplemental method references:**

1 Wei, X. *et al.* MetPP: a computational platform for comprehensive two-dimensional gas chromatography time-of-flight mass spectrometry-based metabolomics. *Bioinformatics* **29**, 1786-1792, doi:10.1093/bioinformatics/btt275 (2013).

2 Caporaso, J. G. *et al.* Ultra-high-throughput microbial community analysis on the Illumina HiSeq and MiSeq platforms. *ISME J* **6**, 1621-1624, doi:10.1038/ismej.2012.8 (2012).

3 Huda, M. N. *et al.* Stool microbiota and vaccine responses of infants. *Pediatrics* **134**, e362-372, doi:10.1542/peds.2013-3937 (2014).

4 Bolyen, E. *et al.* QIIME 2: Reproducible, interactive, scalable, and extensible microbiome data science. Report No. 2167-9843, (PeerJ Preprints, 2018).

5 Quast, C. *et al.* The SILVA ribosomal RNA gene database project: improved data processing and web-based tools. *Nucleic acids research* **41**, D590-D596 (2012).
